# Optimizing the Management of Conduction Disturbances After Self-Expanding TAVR: A Step in the Right Direction

**DOI:** 10.1016/j.jscai.2023.101250

**Published:** 2023-11-27

**Authors:** Giorgio A. Medranda, Suzanne J. Baron

**Affiliations:** a Division of Cardiology, Department of Medicine, NYU Langone Hospital—Long Island, Mineola, New York; b Department of Cardiology, Massachusetts General Hospital, Boston, Massachusetts

**Keywords:** conduction disturbances, pacemaker, transcatheter aortic valve replacement

Over the past decade, the field of transcatheter aortic valve replacement (TAVR) has continued to advance, led predominantly by improvements in transcatheter heart valve (THV) design and improvements in implantation techniques, leading to a progressive decrease in the rates of periprocedural complications.^1^^,^^2^ Nevertheless, conduction disturbances (CD) remain among the more frequent complications following TAVR and have been associated with an increase in heart failure hospitalizations and all-cause mortality.^3^ Although distinct determinants for permanent pacemaker (PPM) implantation following TAVR have been identified, including preexisting CD, short membranous septum length, degree of left ventricular outflow tract calcium, THV type, and depth of implantation^4^^,^^5^, there exists significant institutional variation about when to proceed with PPM implantation,^3^ and the ideal post-TAVR CD management pathway is yet to be determined.

In this issue of the *JSCAI*, Grubb et al^6^ present results from an interim analysis of the Optimize PRO study, in which they examined the Optimize PRO postprocedural CD management algorithm in 400 patients who underwent TAVR using the CoreValve Evolut PRO or PRO+ platform (Medtronic).^6^ Patients were dichotomized based on development of new CD (defined as new-onset left bundle branch block, electrocardiogram [ECG] changes with baseline CD, or periprocedural high-grade atrioventricular block) on 2-hour post-TAVR ECG. For those in the new CD cohort, temporary pacemakers remained in place and ECG assessment was repeated at the 24-hour mark for resolution. Patients with persistent CD after 48 hours either underwent an electrophysiology study (EPS) and were discharged with 30-day continuous ECG monitoring or underwent PPM implantation per institutional discretion. Although PPM requirements were noted to be significantly higher (28.1% vs 1.5%; *P* < .001) in the CD cohort, the investigators’ primary composite safety outcome of all-cause mortality or stroke at 30 days occurred at similar rates between the cohorts (2.4% vs 4.4%; *P* = .334), with no patients suffering sudden cardiac death in either cohort at 30 days. The authors conclude that the Optimize PRO postprocedural CD management algorithm may provide an effective approach to improve PPM utilization in TAVR and facilitate safe monitoring following discharge.

Although it is encouraging that the safety of the Optimize PRO algorithm has been demonstrated in this modestly sized cohort, broad adoption of the pathway may be slow. First, there is likely a learning curve associated with attaining the optimal self-expanding valve position in which the risk of CD is lowest. Indeed, THV implantation remained significantly deeper at the noncoronary cusp in the CD cohort (4.1 mm vs 2.6 mm, *P* > .001) despite a significantly increased rate of resheathing, full recapture, and procedure time, all of which would suggest more attempts at precise and shallow deployment. Implanters will need to commit to the technique and not become dissuaded by the longer procedure times that may be required to optimize valve placement. Whether this learning curve may be accelerated with use of the CoreValve Evolut FX System is yet to be fully elucidated. Additionally, TAVR implanters carry a healthy fear of sudden cardiac death due to progressive CD in the short term following TAVR. Certainly, some of this risk can be mitigated with the placement of 14- to 30-day cardiac rhythm monitors at discharge, but even if all patients received outpatient rhythm monitoring (and not all do), the fear remains that discovery of the CD may be too late and result in patient death. Therefore, there remains a substantial incentive for many institutions to perform EPS and/or implant PPMs prior to discharge to avoid this type of catastrophic and seemingly avoidable complication.

Given the known variations in institutional practices, a uniform TAVR preprocedural, intraprocedural, and postprocedural care pathway that is proven to optimize outcomes in patients receiving different types of THV platforms is truly needed. In terms of management of expected and actualized CD, there have been multiple expert consensus documents that have been developed to offer generalized guidance to TAVR centers^7^^,^^8^; however, these algorithms likely require frequent iterative updates. In addition to the rapid advances in technology and procedural practices, research continues to expand with regard to evaluation of risk for CD after TAVR. A study of 284 patients found that utilizing a protocol of rapid atrial pacing to assess for Wenckebach atrioventricular block after TAVR had a negative predictive value for PPI at 30 days of 98.7%.^9^ Most recently, researchers found that prolonged HV intervals, AH intervals, and Wenckebach cycle lengths were associated with the development of complete heart block after discharge in 399 consecutive patients who underwent an EPS before and after TAVR.^10^ How these findings and others will be adopted into expert guidance pathways as well as into real-world practice remains to be seen.

As the eligible population for TAVR continues to grow with the treatment of low-risk patients and the study of new indications for these devices, efforts to improve THV design/deployment and post-TAVR management have continued to accelerate. Ultimately, robust data will be needed to clarify the ideal post-TAVR CD management algorithm, alter the guidelines, and promote broad adaptation, and the Optimize PRO pathway represents a step in the right direction.
